# The combination of cardiorespiratory fitness and muscular fitness, and prevalence of diabetes mellitus in middle-aged and older men: WASEDA’S Health Study

**DOI:** 10.1186/s12889-022-12971-x

**Published:** 2022-03-30

**Authors:** Dong Wang, Susumu S. Sawada, Hiroki Tabata, Ryoko Kawakami, Tomoko Ito, Kumpei Tanisawa, Mitsuru Higuchi, Kaori Ishii, Koichiro Oka, Katsuhiko Suzuki, Shizuo Sakamoto

**Affiliations:** 1 Graduate School of Sport Sciences, Waseda University, 2-579-15 Mikajima, Tokorozawa, Saitama 359-1192 Japan; 2 Faculty of Sport Sciences, Waseda University, 2-579-15 Mikajima, Tokorozawa, Saitama 359-1192 Japan; 3 Waseda Institute for Sport Sciences, 2-579-15 Mikajima, Tokorozawa City, Saitama, 359-1192 Japan; 4 Sportology Center, Juntendo University Graduate School of Medicine, 2-15-8 Hongo, Bunkyo-ku, Tokyo, Japan; 5 Department of Food and Nutrition, Faculty of Home Economics, Tokyo Kasei University, Tokyo, 173-8602 Japan

**Keywords:** Aerobic capacity, Muscle strength, Muscle mass, Physical activity, Epidemiology

## Abstract

**Background:**

Although the negative relationship between cardiorespiratory fitness (CRF) or muscular fitness and diabetes mellitus were respectively observed in many previous studies, there is still a lack of studies that include CRF and muscular fitness simultaneously. Therefore, this study aimed to investigate the relationship between the combination of CRF and muscular fitness and diabetes through a cross-sectional study.

**Methods:**

This study was part of WASEDA'S Health Study, a cohort study launched in 2014. We used a part of the baseline data collected for this study. Maximal exercise test using a cycle ergometer and leg extension power (LEP) test were respectively used to evaluate CRF and muscular fitness. Since LEP is affected by body weight, relative LEP (rLEP) which is LEP per body weight, was used as an index of muscular fitness. 796 men (56.5 ± 10.4 years old) who completed a medical examination and fitness tests, were divided into two groups based on CRF and rLEP, respectively. The prevalence of diabetes was collected based on a self-reported questionnaire or blood test. Odds ratios and 95% confidence intervals (CIs) for the prevalence of diabetes were obtained using logistic regression models while adjusting for age, body mass index, exercise habits, family history of diabetes, smoking habits, and drinking habits.

**Results:**

55 (7%) participants had diabetes. Compared to participants with lower CRF or rLEP, the odds ratio (95% CIs) of diabetes in those with higher CRF or rLEP was 0.46 (0.21–0.98) or 0.34 (0.16–0.74), respectively. Furthermore, using the lower CRF and lower rLEP group as the reference, the odds ratio (95% CIs) for the lower CRF and higher rLEP group was 0.32 (0.12–0.88), and higher CRF and higher rLEP group was 0.21 (0.07–0.63), after adjusting for potential confounding factors.

**Conclusions:**

CRF and rLEP have independent and joint inverse associations with diabetes prevalence. In addition, participants with high CRF and high rLEP had a lower prevalence of diabetes compared to those with only high CRF or only high rLEP.

**Supplementary Information:**

The online version contains supplementary material available at 10.1186/s12889-022-12971-x.

## Background

The number of individuals with diabetes mellitus in the world has increased significantly from 108 million in 1980 to 422 million in 2014, while the prevalence of diabetes among adults aged 18 and older has risen from 4.7% in 1980 to 8.5% in 2014 [1]. The number of people with diabetes is projected to rise to 700 million (10.9%) by 2045 [2].

In 2012, 1.5 million people died as a direct result of diabetes and hyperglycemia increases the risk of cardiovascular and other diseases, resulting in the death of 2.2 million people [1]. Out of a total of 3.7 million people, 43% died before reaching the age of 70 [1]. World Health Organization estimates that diabetes is one of the top 10 causes of death in 2019 [3]. Diabetes is an important public health issue in the world, not only because of its health impact but also because of the economic burden it imposes [1].

Many observational studies have reported that there is a negative relationship between cardiorespiratory fitness (CRF) and diabetes [4–10]. These studies show that the relationship between physical activity and diabetes is independent of ethnicity, and that individuals with lower levels of physical activity have higher risk of developing type 2 diabetes. Similarly, a number of observational studies have reported a negative relationship between muscular strength or muscle mass, an index of muscular fitness, and diabetes [11–21]. These studies suggest that the prevalence of diabetes in individuals with high CRF and muscular fitness may be lower than individuals with high CRF alone or individuals with higher muscular fitness alone. However, there are no studies that have investigated the relationship between the combination of CRF and muscular fitness, and diabetes. Therefore, this study investigated the relationship between the combination of CRF and leg extension power (LEP) as an index of muscular fitness and diabetes through a cross-sectional study.

## Methods

### Participants

This is a cross-sectional study using the baseline survey data in WASEDA’S Health Study (Waseda Alumni's Sports, Exercise, Daily Activity, Sedentariness and Health Study). WASEDA’S Health Study is a cohort study conducted with Waseda University’s alumni and their spouses aged 40 or older to examine the relationship between their health outcomes and their habits of sports, exercises, physical activities and sedentary behavior.

We published the overview and objectives of the study through its alumni association, which includes approximately 660 000 people, to invite participants. WASEDA’S Health Study consists of four cohorts (Cohort A to D). For participants in Cohort A, a survey about their physical activities and health outcomes was conducted through the online self-administered questionnaire. For participants in Cohort B, measurements of the amount of their physical activities and the hours of their sitting behavior were conducted using an accelerometer, in addition to the online self-administered questionnaire. For participants in Cohort C, several medical tests were conducted, in addition to Cohort B’s survey items. For participants in Cohort D, physical fitness tests and more detailed medical tests were conducted, in addition to Cohort C’s survey items. Alumni who wanted to join WASEDA'S Health Study could choose any course from Cohort A to D. The participants in Cohort D were chosen as the participants of this study.

The participants in this study were 1387 individuals who joined the Cohort D survey of WASEDA’S Health Study from March 2015 to March 2020 and performed the maximal exercise test and LEP measurement as baseline measurements. The participants include those taking medication due to hypertension, diabetes, and dyslipidemia. Several exclusion criteria were established for accurate analysis. First, those who could not perform the LEP measurement or whose data were clearly abnormal (*n* = 7), those who had consumed any food in the morning of the measurement (*n* = 12), and those who had missing blood test data (*n* = 7) were excluded from the analysis. Then, we also excluded those with missing data of self-administered questionnaire (*n* = 3), CRF (*n* = 10), leg circumference (*n* = 52), grip strength (*n* = 6), and body fat percentage (*n* = 1). Those with a history of heart disease (*n* = 66) were also excluded. Women (*n* = 427) were excluded because the number of female diabetes patients was small (*n* = 6), thus only men were included in the analysis of this study (*n* = 796).

Before the baseline survey, all participants received an explanation of the study and provided their written informed consent. This study has been approved by the research ethics committee of Waseda University (approval number: 2014-G002 and 2018-G001). The report was prepared in accordance with the Strengthening the Reporting of Observational Studies in Epidemiology (STROBE) reporting guidelines for cohort studies.

### Measures

#### Medical tests

We conducted a survey on the participants’ sex, age, exercise habits, family history of diabetes, drinking habits, and smoking habits by using a self-administered questionnaire. Exercise habits were categorized into two groups: “with exercise habit” and “without exercise habit”; smoking habits were categorized into three groups: “never smoker”, “former smoker”, and “current smoker”; and drinking habits were classified into four groups: “non-drinker”, “ ≤ 1 day/week”, “2 − 3 days/week”, and “ ≥ 4 days/week”.

A stadiometer and body composition analyzer (MC-980A; Tanita Corp., Tokyo, Japan) were used to measure their height, weight, and body fat percentage. Body weight was measured with participants removing their shoes and wearing light clothing. Based on the results, body mass index was determined. The waist circumference and calf circumference were measured with a measuring tape. The grip strength was also measured using a digital grip strength tester (T.K.K.5401; Takei Scientific Instruments Co., Ltd., Niigata, Japan).

The participants were instructed to fast from the night before the blood test. Blood collection was performed prior to the physical fitness tests. We collected the participants’ venous blood from their forearm vein after making sure that they did not have breakfast. The survey items used for this study include the items related to fat metabolism (total cholesterol, HDL cholesterol, LDL cholesterol, triglycerides) and glucose metabolism (fasting blood glucose, HbA1c). The blood pressure at rest was measured based on the Japanese Society of Hypertension Guidelines for the Management of Hypertension [22] using automated sphygmomanometer (HEM-7250-IT; Omron Healthcare Co., Ltd., Kyoto, Japan).

#### Cardiorespiratory fitness

All participants performed a maximal exercise test on a cycle ergometer (828E; Monarch Exercise AB, Kingdom of Sweden) to determine the peak oxygen uptake as an index of CRF. After recording an electrocardiogram (ML-9000 and MLX-1000; Fukuda Denshi Co., Ltd., Tokyo, Japan) for three minutes at rest, the participants started the exercise test (men starting from 30 W, women starting from 15 W), increasing 15 W every minute until they were exhausted. They were wearing a mask for respiratory gas analysis during the exercise test and expired gases, pulmonary VO_2_, and VCO_2_, by a breath-by-breath method using an automated gas analysis system were analyzed (AE310S and AE100i; Minato Medical Science Co., Ltd., Osaka, Japan). A blood pressure cuff was also attached to the left upper arms to evaluate blood pressure using an automatic blood pressure monitor for the exercise test (Tango M2; Sun Tech Medical Inc., Morrisville, North Carolina, USA). Heart rate and blood pressure were used for safety control before and during the exercise. The endpoint of the exercise test was when their heart rate reached approximately 90% of the predicted maximum heart rate by age, achieving a respiratory exchange ratio > 1.1, and rating of perceived exertion of ≥ 18 or plateauing oxygen intake [23]. We determined that the test was valid if participants had achieved three of the four criteria. The exercise test was stopped when the participants expressed an inability to continue exercise; or when the systolic blood pressure reached 250 mmHg. After the exercise, the participants had a one-minute recovery time and a two-minute rest in the sitting position to conclude the test. Then the highest value of the average oxygen intake at intervals of 30 s during exercise was defined as peak oxygen uptake.

#### Leg extension power

LEP was measured using a LEP measurement machine that applied a brake load (AnaeroPress 3500; Combi Corp., Tokyo, Japan) during the measurement, the patients were sitting deeply in the chair so that the gap between the waist and back was as narrow as possible, with both feet placed on the foot plate. The chair position was adjusted so that the knee angle was 90 degrees. Their waist and both legs were secured with belts respectively. Five leg extension movements were performed at full force with a load value equal to the body weight of the participants. The participants were instructed to grip the levers on both sides and kick as hard and fast as they could. Calibration (bending and stretching of the legs twice) was performed between each trial, at which time we checked whether the legs were fully extended and whether the waist position shifted. The maximum value of five trials was used for data analysis. Since LEP is affected by body weight, relative LEP (rLEP) which is LEP per body weight (W/kg), was used as an index of muscular fitness.

### Determination of diabetes

We defined the participants who answered that they had diabetes in the self-administered questionnaire conducted during the baseline survey as diabetics. In addition, in accordance with the Japan Diabetes Society guidelines, the participants with fasting blood glucose level of 126 mg/dL (7.0 mmol/L) or above in the baseline survey, or with HbA1c of 6.5% or above were also defined as diabetics [24].

### Statistics analysis

In order to ascertain the physical characteristics of the study participants, we classified the participants into two groups of diabetics and non-diabetics, and the physical characteristics of both groups were compared. The study participants were divided into two groups by the median of CRF and rLEP, and the physical characteristics of the low CRF group (low CRF), high CRF group (high CRF), the low rLEP group (low rLEP), and the high rLEP group (high rLEP) were compared. The group with low CRF and low rLEP was designated as "low CRF & low rLEP", the group with low CRF and high rLEP was designated as "low CRF & high rLEP", the group with high CRF and low rLEP was designated as "high CRF & low rLEP", and the group with high CRF and high rLEP was designated as "high CRF & high rLEP " for comparison of physical characteristics.

A logistic regression model was used to evaluate how CRF and rLEP each related to the prevalence of diabetes after adjusting for potential confounding factors. First, the relationship between CRF and the prevalence of diabetes was analyzed by inputting the presence of diabetes as the dependent variable and CRF as the independent variable. Factors adjusted using a logistic regression model were age (continuous), body mass index (continuous), exercise habits (yes, no), family history of diabetes (yes, no), smoking habits (current smoker, former smoker, never smoker), and drinking habits (non-drinker, ≤ 1 day/week, 2 − 3 days/week, ≥ 4 days/week). Next, the relationship between rLEP and the prevalence of diabetes was analyzed using a logistic regression model with the presence of diabetes as the dependent variable and rLEP as the independent variable. In addition, we conducted an analysis to determine the relationship between the combination of CRF and rLEP and the prevalence of diabetes. IBM SPSS Statistics 26 (IBM Corp., Armonk, NY, USA) was used for analysis.

## Results

### Characteristics of the study participants

The mean age ± standard deviation of the participants was 56.5 ± 10.4 years old. Among them, 55 (7%) were diabetics.

Table 1 shows participants’ physical characteristics according to the presence or absence of diabetes. With regard to continuous variables, the diabetic group tended to be older, have a larger waist circumference, and have higher systolic blood pressure and triglycerides. In addition, the diabetic group tended to have lower CRF. With regard to categorical variables, the diabetic group tended to have higher rates of hypertension and dyslipidemia. As for smoking and drinking habits, the diabetic group tended to have a higher percentage of smokers and non-drinkers. The diabetic group showed a higher tendency of respondents who answered that they had exercise habits.

**Table 1:** Characteristics of study participants according to diabetes status

	Total (*n* = 796)	Diabetes (*n* = 55)	No diabetes (*n* = 741)
Age (years)	56.5 ± 10.4	64.0 ± 9.1	55.9 ± 10.2
Height (cm)	170.3 ± 5.8	169.3 ± 5.0	170.4 ± 5.8
Weight (kg)	68.7 ± 9.7	69.7 ± 10.8	68.6 ± 9.7
Waist circumference (cm)	84.5 ± 8.6	87.7 ± 9.0	84.3 ± 8.5
Calf circumference (cm)	37.5 ± 2.6	37.3 ± 3.2	37.5 ± 2.5
Body mass index (kg/m^2^)	23.7 ± 3.0	24.3 ± 3.4	23.6 ± 3.0
Percent body fat (%)	20.5 ± 5.8	23.2 ± 5.3	20.3 ± 5.8
Systolic blood pressure (mmHg)	132.0 ± 18.8	139.3 ± 20.9	131.4 ± 18.5
Diastolic blood pressure (mmHg)	83.5 ± 11.6	84.1 ± 13.3	83.4 ± 11.4
Grip strength (kg)	36.8 ± 5.7	34.4 ± 6.2	37.0 ± 5.6
Relative grip strength, (kg/kg)	0.54 ± 0.09	0.50 ± 0.08	0.55 ± 0.09
Leg extension power (W)	1278 ± 368	1088 ± 348	1292 ± 366
Relative leg extension power (W/kg)	18.6 ± 4.8	15.5 ± 3.9	18.9 ± 4.8
Peak oxygen uptake (mL/kg/min)	30.3 ± 6.4	26.1 ± 4.6	30.6 ± 6.4
Total cholesterol (mg/dL)	209.9 ± 34.1	202.4 ± 33.6	210.4 ± 34.1
HDL cholesterol (mg/dL)	61.9 ± 16.0	57.7 ± 18.2	62.2 ± 15.8
LDL cholesterol (mg/dL)	122.9 ± 29.8	118.2 ± 29.8	123.2 ± 29.8
Triglycerides (mg/dL)^a^	87.0 (62.0 − 127.0)	95.0 (68.0 − 151.0)	87.0 (61.0 − 126.5)
Fasting glucose (mg/dL)	98.2 ± 14.5	133.4 ± 25.9	95.6 ± 8.8
HbA1c (%)	5.5 ± 0.5	6.7 ± 0.7	5.4 ± 0.3
Exercise habit (%)	541 (68.0)	42 (76.4)	499 (67.3)
Hypertension (%)	188 (23.6)	20 (36.4)	168 (22.7)
Dyslipidemia (%)	205 (25.8)	24 (43.6)	181 (24.4)
Family history of diabetes (%)	182 (22.9)	20 (36.4)	162 (21.9)
Family history of hypertension (%)	350 (44.0)	26 (47.3)	324 (43.7)
Family history of dyslipidemia, (%)	82 (10.3)	6 (10.9)	76 (10.3)
Current smokers (%)	63 (7.9)	7 (12.7)	56 (7.6)
Non-drinkers (%)	103 (12.9)	10 (18.2)	93 (12.6)

Data are express as means ± standard deviation or percentages of participants

^a^Median (Interquartile range)

Table 2 shows participants’ physical characteristics according to the combination of CRF and rLEP (Supplemental File 1: Table A shows characteristics of study participants according to CRF and rLEP). With regard to continuous variables, the low CRF & low rLEP group tended to be older, have a larger waist circumference, and show higher body fat percentage and blood pressure compared to the other groups. On the other hand, the low CRF & low rLEP group and the low CRF & high rLEP group tended to have lower HDL cholesterol and higher triglycerides. With regard to categorical variables, the low CRF & low rLEP group and the low CRF & high rLEP group had a lower percentage of respondents who answered that they had an exercise habit. In addition, the low CRF & low rLEP group tended to have a higher percentage of hypertension prevalence. In terms of smoking habits, the high CRF & high rLEP group had a lower percentage of smokers. In terms of drinking habits, the low CRF & high rLEP group tended to have a higher percentage of non-drinkers.

**Table 2:** Characteristics according to combined cardiorespiratory fitness and relative leg extension power

	Low CRF & Low rLEP (*n* = 269)	Low CRF & High rLEP (*n* = 129)	High CRF & Low rLEP (*n* = 129)	High CRF & High rLEP (*n* = 269)
Age (years)	63.1 ± 9.8	56.6 ± 9.1	55.6 ± 9.3	50.1 ± 7.5
Height (cm)	169.6 ± 6.0	170.5 ± 5.9	170.5 ± 5.9	171.0 ± 5.4
Weight (kg)	71.2 ± 11.0	69.5 ± 8.4	66.6 ± 8.7	66.8 ± 8.8
Waist circumference (cm)	89.1 ± 8.7	85.3 ± 7.0	81.9 ± 7.5	80.8 ± 7.3
Calf circumference (cm)	37.7 ± 2.9	37.4 ± 2.6	37.5 ± 2.4	37.4 ± 2.3
Body mass index (kg/m^2^)	24.7 ± 3.4	23.9 ± 2.9	22.9 ± 2.6	22.8 ± 2.5
Percent body fat (%)	23.9 ± 5.5	20.8 ± 4.8	18.4 ± 5.1	18.0 ± 5.0
Systolic blood pressure (mmHg)	138.6 ± 19.9	133.4 ± 17.3	129.3 ± 17.2	125.9 ± 16.8
Diastolic blood pressure (mmHg)	85.6 ± 12.1	85.6 ± 11.0	81.9 ± 9.9	81.1 ± 11.4
Grip strength (kg)	34.7 ± 5.8	38.0 ± 5.9	36.4 ± 4.5	38.7 ± 5.3
Relative grip strength, (kg/kg)	0.49 ± 0.08	0.55 ± 0.10	0.55 ± 0.07	0.58 ± 0.08
Leg extension power (W)	1033 ± 273	1484 ± 273	1041 ± 222	1537 ± 305
Relative leg extension power (W/kg)	14.4 ± 2.8	21.3 ± 2.3	15.6 ± 2.5	23.0 ± 3.4
Peak oxygen uptake (mL/kg/min)	24.7 ± 3.0	26.4 ± 2.5	33.5 ± 3.3	36.1 ± 5.2
Total cholesterol (mg/dL)	209.2 ± 36.1	208.5 ± 32.8	212.1 ± 31.8	210.3 ± 33.8
HDL cholesterol (mg/dL)	58.5 ± 14.9	59.1 ± 13.1	66.2 ± 16.9	64.5 ± 16.9
LDL cholesterol (mg/dL)	124.0 ± 31.9	121.6 ± 28.3	123.4 ± 29.7	122.1 ± 28.6
Triglycerides (mg/dL)^a^	99.0 (69.0 − 141.0)	103.0 (73.0 − 151.5)	77.0 (55.0 − 106.0)	79.0 (55.5 − 114.0)
Fasting glucose (mg/dL)	102.7 ± 16.9	97.4 ± 12.1	95.6 ± 13.0	95.3 ± 12.3
HbA1c (%)	5.6 ± 0.6	5.5 ± 0.5	5.4 ± 0.4	5.3 ± 0.4
Exercise habit (%)	158 (58.7)	79 (61.2)	99 (76.7)	205 (76.2)
Hypertension (%)	105 (39.0)	29 (22.5)	29 (22.5)	25 (9.3)
Dyslipidemia (%)	80 (29.7)	38 (29.5)	36 (27.9)	51 (19.0)
Family history of diabetes (%)	66 (24.5)	30 (23.3)	24 (18.6)	62 (23.0)
Family history of hypertension (%)	128 (47.6)	59 (45.7)	56 (43.4)	107 (39.8)
Family history of dyslipidemia (%)	28 (10.4)	13 (10.1)	12 (9.3)	29 (10.8)
Current smokers (%)	21 (7.8)	13 (10.1)	11 (8.5)	18 (6.7)
Non-drinkers (%)	37 (13.8)	20 (15.5)	13 (10.1)	33 (12.3)
Diabetes (%)	38 (14.1)	5 (3.9)	7 (5.4)	5 (1.9)

*CRF* cardiorespiratory fitness, *rLEP* relative leg extension power

Data are express as means ± standard deviation or percentages of participants

^a^Median (Interquartile range)

### Diabetes prevalence by CRF and rLEP

Table 3 shows the multivariable-adjusted odds ratios and 95% confidence intervals (CIs) of the prevalence of diabetes by CRF and rLEP respectively. In terms of the prevalence of diabetes by CRF, which were classified into two groups by median, the odds ratio (95% CI) for the high CRF group compared with the low CRF group was 0.46 (0.21–0.98) after adjustment for potential confounding factors, which had a significantly low odds ratio. In terms of the prevalence of diabetes by rLEP, the odds ratio (95% CI) for the high rLEP group compared to the low rLEP group was 0.34 (0.16–0.74) after adjustment for potential confounding factors, indicating a significant negative relationship.

**Table 3:** Odds ratios for prevalence of diabetes mellitus according to cardiorespiratory fitness and relative leg extension power

	*n*	Cases	Cases per 1000	Unadjusted OR (95% CI)	Age-adjusted OR (95% CI)	Multivariable-adjusted OR^a^ (95% CI)
Cardiorespiratory fitness						
Low cardiorespiratory fitness	398	43	108	1.00 (reference)	1.00 (reference)	1.00 (reference)
High cardiorespiratory fitness	398	12	30	0.26 (0.13 − 0.50)	0.45 (0.22 − 0.92)	0.46 (0.21 − 0.98)
Relative leg extension power						
Low relative leg extension power	398	45	113	1.00 (reference)	1.00 (reference)	1.00 (reference)
High relative leg extension power	398	10	25	0.20 (0.10 − 0.41)	0.33 (0.16 − –0.69)	0.34 (0.16 − 0.74)

*OR* odds ratio, *CI* confidence interval

^a^Adjusted for age (continuous variable), body mass index (continuous variable), exercise habit (yes, no), family history of diabetes (yes, no), smoking habits (never smoker, former smoker, or current smoker), drinking habits (non-drinker, ≤ 1 day/week, 2 − 3 days/week, ≥ 4 days/week)

### Diabetes prevalence by the combination of CRF and rLEP

Table 4 shows the odds ratios and 95% CIs for the combination of CRF and rLEP and the prevalence of diabetes. After adjustment for potential confounders, the odds ratio (95% CI) for the low CRF & high rLEP group was 0.32 (0.12–0.88), while the odds ratio for the high CRF & high rLEP group was 0.21 (0.07–0.63), which were significantly low odds ratios. The odds ratio for the high CRF & low rLEP group was low, but not statistically significant.

**Table 4:** Odds ratios for prevalence of diabetes mellitus according to combined cardiorespiratory fitness and relative leg extension power

	*n*	Cases	Cases per 1000	Unadjusted OR (95% CI)	Age-adjusted OR (95% CI)	Multivariable-adjusted OR^a^ (95% CI)
Low CRF & Low rLEP	269	38	141	1.00 (reference)	1.00 (reference)	1.00 (reference)
Low CRF & High rLEP	129	5	39	0.25 (0.09 − 0.64)	0.33 (0.12 − 0.88)	0.32 (0.12 − 0.88)
High CRF & Low rLEP	129	7	54	0.35 (0.15 − 0.80)	0.49 (0.21 − 1.18)	0.46 (0.18 − 1.16)
High CRF & High rLEP	269	5	19	0.12 (0.05 − 0.30)	0.22 (0.08 − 0.61)	0.21 (0.07 − 0.63)

*OR* odds ratio, *CI* confidence interval, *CRF* cardiorespiratory fitness, *rLEP* relative leg extension power

^a^Adjusted for age (continuous variable), body mass index (continuous variable), exercise habit (yes, no), family history of diabetes (yes, no), smoking habits (never smoker, former smoker, or current smoker), drinking habits (non-drinker, ≤ 1 day/week, 2 − 3 days/week, ≥ 4 days/week)

## Discussion

This cross-sectional study investigated the prevalence of diabetes in middle-aged and older men by CRF and rLEP, and the relationship between the combination of CRF and rLEP and the prevalence of diabetes. The main results of this study are as follows. 1) As in previous studies, CRF and rLEP independently had a negative association with the prevalence of diabetes. 2) The combination of CRF and rLEP showed a strong negative association with the prevalence of diabetes.

### Prevalence of diabetes by CRF

The study showed that after multivariable adjustment, the high CRF group had a significantly lower prevalence of diabetes. There are several previous studies that have reported lower diabetes incidence among individuals with higher levels of measured CRF or self-reported physical activity [4–7, 10]. Similarly, Hartaigh et al. investigated the relationship between resting heart rate and the prevalence of diabetes in older Chinese and reported a lower prevalence of diabetes in the group with a lower resting heart rate [8]. Furthermore, CRF was inversely associated with the prevalence of metabolic syndrome in cross-sectional studies [9, 25]. These studies suggest that regular physical activity, or as a result, maintaining high CRF, regardless of ethnicity or gender, may be associated with the prevention of diabetes.

One possible mechanism behind this theory is that aerobic exercise uses fat as the main source of energy to reduce adipose tissue mass, decrease resistin concentrations and increase adiponectin secretion, which results in improved insulin resistance [26].

### Prevalence of diabetes by rLEP

This study observed that there was a negative relationship between rLEP and the prevalence of diabetes. The group with high rLEP had a significantly lower odds ratio for the prevalence of diabetes.

Although fewer in number than CRF, there are some previous studies that have examined the association between muscle strength or muscle mass and diabetes [11–20]. For instance, serval studies have shown that low grip strength is associated with higher prevalence and incidence [13, 19, 20]. Mainous et al. reported that grip strength in diabetics was lower than in non-diabetics among Americans (body mass index: 18.5 − 25 kg/m^2^) [18]. Also, diabetics had lower muscle quality and more reduced strength in the lower limbs compared to people without diabetes among middle-aged and older people [15–17]. In addition, Momma et al. reported that lower relative grip strength was associated with a higher incidence of diabetes [13]. Furthermore, Sawada et al. investigated the relationship between muscular power and muscular endurance with the incidence of diabetes in Japanese men. They showed that there was a significant relationship between muscular endurance and incidence of diabetes, however no relationship with muscular power [12]. Wang et al. reported that moderate muscular strength, but not upper muscular strength, was associated with a reduced risk of developing diabetes independent of estimated CRF [21]. These studies suggest that maintaining high or a threshold level of muscle strength, as is the case in CRF, may be associated with the prevention of diabetes regardless of ethnicity or gender.

One possible mechanism behind this theory is that resistance exercise increases muscle mass, muscle metabolic function, and the function of muscle glycolytic system and other insulin signaling systems in muscle such as insulin receptor substrate 1 and glucose transporter type 4 (GLUT4) [27].

### Prevalence of diabetes by the combination of CRF and rLEP

The relationship between physical fitness indicators and prevalence of diabetes in this study suggests that although the higher rLEP may have more benefits on the prevention of diabetes than CRF, the simultaneous enhancement of rLEP and CRF may contribute more than promoting rLEP alone. Similarly, Jurca et al. suggested that the combination of higher muscle strength and higher CRF may have more benefits than only improving one of fitness on the preventing the metabolic syndrome, thereby preventing the diabetes, despite this study based on American man indicated that the higher CRF may be more beneficial than higher muscle strength, which was a little different from our results [25]. This inconsistency may due to the differences of sample size, race and measurement methods. In a cohort study of American women, Grontved et al. investigated the relationship between various physical activity patterns and incidence of diabetes, and not only aerobic physical activity but also muscle-strengthening and conditioning activities could contribute to diabetes prevention [28]. It is well established that aerobic physical activity can lower the risk of diabetes, however other types of physical activity may also contribute to diabetes prevention, and it is considered desirable to be active in all aspects of life for diabetes prevention. Besides, it is necessary to clarify what kind as well as how much physical activity contributes to diabetes prevention in a future study.

One possible mechanism behind this theory is that exercise activates AMP-activated protein kinase (AMPK), which regulates glucose and lipid metabolism [29, 30]. In addition, activated AMPK and Ca^2+^ released by muscle contraction may induce translocation of GLUT4 via Rab GTPase-activating proteins Tre-2/BUB2/cdc 1 domain family (TBC1D) 1 and TBC1D4, thereby promoting glucose uptake in skeletal muscle [29, 30].

Since both aerobic exercise and resistance exercise promote these functions, the effects of both may be integrated to be more effective in preventing diabetes.

### Strengths and limitations

This study used not only self-reports but also blood tests in determining diabetes, which is considered to be an accurate way to discriminate for diabetes. In addition, another strength of this study is that both CRF and rLEP were measured objectively using direct measurement.

This study has some limitations. Firstly, this study is a cross-sectional study and not designed to refer to any causal relationship. Secondly, the participants of this study were only graduates of Waseda University and their spouses, which is mainly a highly educated Japanese population. The prevalence of diabetes among the participants in this study was 5%, which was lower than the 11.8% of the prevalence of diabetes among the men in Japan reported by the World Health Organization [31]. This could be attributed to the fact that the participants in this study were those who voluntarily participated in WASEDA'S Health Study and were highly health conscious. The percentage of participants with exercise habits was 60.7% for women and 68.0% for men, which was clearly higher than the percentage of participants with exercise habits in the National Health and Nutrition Survey (25.1% for women and 33.4% for men) [32]. However, 99.8% of the participants in this study were Japanese and they did not have characteristics that differed significantly from the average Japanese of the same age except for the percentage of those with exercise habits. Therefore, the results of this study can represent the Japanese or East Asian characteristics to some extent. In addition, this study is only for those who wished to participate in the study, and there may be volunteer bias. Thirdly, there is a possibility that some of the associations are explained by genetic confounding. However, adjusting the family history of diabetes did not dramatically change the associations, suggesting that there was no strong effect of the gene on the associations. Fourthly, in exercise habits, only the presence or absence of exercise habits was investigated, and exercise intensity and frequency could not be taken into account. Finally, nutritional intake was not considered in this study. Future prospective studies are required in diverse populations of men and women to further understand the joint and independent role of CRF and muscle strength in the prevention of diabetes.

## Conclusions

CRF and rLEP in men showed a statistically significant negative relationship with the prevalence of diabetes. Those with high CRF and high muscle strength had a lower prevalence of diabetes compared to those with only high CRF or only high muscle strength. Although the cross-sectional study makes it difficult to make a definitive assessment of causality, maintaining and improving both CRF and muscle strength may prevent or delay the onset of diabetes in middle-aged and older people.

## Supplementary Information

**Additional file 1:** **Table A.** Characteristics of study participants according to cardiorespiratory fitness and relative leg extension power.

## Data Availability

The datasets used and/or analyzed during the current study are available from the corresponding author on reasonable request.

## Abbreviations

CRF: Cardiorespiratory fitness
LEP: Leg extension power
rLEP: Relative Leg extension power
CI: Confidence interval
