# Supplementary material for: The combination of cardiorespiratory fitness and muscular fitness, and prevalence of diabetes mellitus in middle-aged and older men: WASEDA’S Health Study
Source: BMC Public Health. 2022 Mar 30;22:626. doi: 10.1186/s12889-022-12971-x (PMC8969323; doi:10.1186/s12889-022-12971-x)
Supplement: Supplementary file 1 — Additional file 1: Table A. Characteristics of study participants according to cardiorespiratory fitness and relative leg extension power. [file 12889_2022_12971_MOESM1_ESM.docx]

Table A Characteristics of study participants according to cardiorespiratory fitness and relative leg extension power

|  | Low CRF  (*n* = 398) | High CRF  (*n* = 398) | Low rLEP  (*n* = 398) | High rLEP  (*n* = 398) |
| --- | --- | --- | --- | --- |
| Age (years) | 61.0 ± 10.1 | 51.9 ± 8.5 | 60.7 ± 10.3 | 52.2 ± 8.6 |
| Height (cm) | 169.9 ± 6.0 | 170.8 ± 5.5 | 169.9 ± 6.0 | 170.8 ± 5.6 |
| Weight (kg) | 70.7 ± 10.3 | 66.7 ± 8.7 | 69.7 ± 10.5 | 67.7 ± 8.7 |
| Waist circumference (cm) | 87.9 ± 8.3 | 81.2 ± 7.4 | 86.8 ± 9.0 | 82.3 ± 7.5 |
| Calf circumference (cm) | 37.6 ± 2.8 | 37.5 ± 2.4 | 37.6 ± 2.7 | 37.4 ± 2.4 |
| Body mass index (kg/m^2^) | 24.5 ± 3.2 | 22.8 ± 2.5 | 24.1 ± 3.2 | 23.2 ± 2.7 |
| Percent body fat (%) | 22.9 ± 5.5 | 18.2 ± 5.0 | 22.1 ± 6.0 | 18.9 ± 5.1 |
| Systolic blood pressure (mmHg) | 136.9 ± 19.2 | 127.0 ± 17.0 | 135.6 ± 19.5 | 128.4 ± 17.3 |
| Diastolic blood pressure (mmHg) | 85.6 ± 11.7 | 81.3 ± 11.0 | 84.4 ± 11.6 | 82.5 ± 11.5 |
| Grip strength (kg) | 35.7 ± 6.0 | 37.9 ± 5.2 | 35.2 ± 5.5 | 38.5 ± 5.5 |
| Relative grip strength, (kg/kg) | 0.51 ± 0.09 | 0.57 ± 0.08 | 0.51 ± 0.08 | 0.57 ± 0.09 |
| Leg extension power (W) | 1179 ± 345 | 1376 ± 365 | 1036 ± 257 | 1520 ± 296 |
| Relative leg extension power (W/kg) | 16.7 ± 4.2 | 20.6 ± 4.7 | 14.8 ± 2.8 | 22.5 ± 3.2 |
| Peak oxygen uptake (mL/kg/min) | 25.3 ± 2.9 | 35.2 ± 4.8 | 27.6 ± 5.1 | 32.9 ± 6.4 |
| Total cholesterol (mg/dL) | 208.9 ± 35.0 | 210.8 ± 33.2 | 210.1 ± 34.7 | 209.7 ± 33.5 |
| HDL cholesterol (mg/dL) | 58.7 ± 14.3 | 65.1 ± 16.9 | 61.0 ± 15.9 | 62.7 ± 16.0 |
| LDL cholesterol (mg/dL) | 123.2 ± 30.7 | 122.5 ± 28.9 | 123.8 ± 31.2 | 121.9 ± 28.5 |
| Triglycerides (mg/dL)^a^ | 100.0 (70.0−142.0) | 79.0 (55.0−111.3) | 91.0 (63.0−129.3) | 85.0 (60.0−125.3) |
| Fasting glucose (mg/dL) | 101.0 ± 15.7 | 95.4 ± 12.5 | 100.4 ± 16.1 | 96.0 ± 12.3 |
| HbA1c (%) | 5.6 ± 0.6 | 5.4 ± 0.4 | 5.6 ± 0.5 | 5.4 ± 0.4 |
| Exercise habit (%) | 237 (59.5) | 304 (76.4) | 257 (64.6) | 284 (71.4) |
| Hypertension (%) | 134 (33.7) | 54 (13.6) | 134 (33.7) | 54 (13.6) |
| Dyslipidemia (%) | 118 (29.6) | 87 (21.9) | 116 (29.1) | 89 (22.4) |
| Family history of diabetes (%) | 96 (24.1) | 86 (21.6) | 90 (22.6) | 92 (23.1) |
| Family history of hypertension (%) | 187 (47.0) | 163 (41.0) | 184 (46.2) | 166 (41.7) |
| Family history of dyslipidemia (%) | 41 (10.3) | 41 (10.3) | 40 (10.1) | 42 (10.6) |
| Current smokers (%) | 34 (8.5) | 29 (7.3) | 32 (8.0) | 31 (7.8) |
| Non-drinkers (%) | 57 (14.3) | 46 (11.6) | 50 (12.6) | 53 (13.3) |
| Diabetes (%) | 43 (10.8) | 12 (3.0) | 45 (11.3) | 10 (2.5) |

*CRF* cardiorespiratory fitness, *rLEP* relative leg extension power

Data are express as means ± standard deviation or percentages of participants

^a^ Median (Interquartile range)

Table A shows participants’ physical characteristics according to CRF and rLEP. With regard to continuous variables, the low CRF group tended to be older, have a larger waist circumference, and have higher body fat percentage, blood pressure, and triglycerides. On the other hand, the low CRF group tended to have lower relative grip strength and rLEP, and lower HDL cholesterol. With regard to categorical variables, the high CRF group had a higher percentage of respondents who answered that they had an exercise habit. In addition, the low CRF group tended to have a higher percentage of hypertension, dyslipidemia, and diabetes prevalence. No clear differences in smoking and drinking habits were observed between the two groups. Furthermore, by rLEP, the low rLEP group tended to be older, have a larger waist circumference, and show higher body fat percentage, blood pressure, and triglycerides than the high rLEP group. On the other hand, the low rLEP group tended to have lower grip strength and CRF. The low rLEP group had a higher percentage of hypertension, dyslipidemia, and diabetes prevalence. No clear differences in smoking and drinking habits were observed between the two groups.
